# ZNF205 positively regulates RLR antiviral signaling by targeting RIG-I: ZNF205 regulates RLR antiviral signaling

**DOI:** 10.3724/abbs.2023136

**Published:** 2023-08-14

**Authors:** Ni Zhong, Chen Wang, Guangxiu Weng, Ting Ling, Liangguo Xu

**Affiliations:** College of Life Science Jiangxi Normal University Nanchang 330022 China

**Keywords:** ZNF205, RIG-I, ubiquitination, RLR signaling

## Abstract

Retinoic acid-inducible gene I (RIG-I) is a cytosolic viral RNA receptor. Upon viral infection, the protein recognizes and then recruits adapter protein mitochondrial antiviral signaling (MAVS) protein, initiating the production of interferons and proinflammatory cytokines to establish an antiviral state. In the present study, we identify zinc finger protein 205 (ZNF205) which associates with RIG-I and promotes the Sendai virus (SeV)-induced antiviral innate immune response. Overexpression of ZNF205 facilitates interferon-beta (IFN-β) introduction, whereas ZNF205 deficiency restricts its introduction. Mechanistically, the C-terminal zinc finger domain of ZNF205 interacts with the N-terminal tandem caspase recruitment domains (CARDs) of RIG-I; this interaction markedly promotes K63 ubiquitin-linked polyubiquitination of RIG-I, which is crucial for RIG-I activation. Thus, our results demonstrate that ZNF205 is a positive regulator of the RIG-I-mediated innate antiviral immune signaling pathway.

## Introduction

The first line of host defense against pathogens, including bacteria and viruses, is the innate immune response. Pathogen-associated molecular patterns (PAMPs), structurally conserved microbial components of invading pathogens, are recognized by host pattern-recognition receptors (PRRs). A number of host cell signaling events are activated upon detection of infection, leading to the introduction of type I interferons (IFNs), including interferon-alpha (IFN-α) and IFN-β family cytokines, proinflammatory cytokines, and other immunomodulatory molecules [ 1‒ 3]. These proteins further mediate and enhance the activation of the adaptive immune system to eliminate pathogens [ 1, 4, 5].

Four distinct classes of PRR families have been identified, including Toll-like receptors (TLRs), RIG-I-like receptors (RLRs), C-type lectin receptors (CLRs), and NOD-like receptors (NLRs) [ 2, 3]. The RLR family consists of three members: RIG-I, melanoma differentiation-associated gene 5 (MDA5), and laboratory of genetics and physiology 2 (LGP2) [6]. All proteins contain a DEAD-box helicase domain which is responsible for RNA binding and ATP hydrolysis, and a C-terminal regulatory domain (CTD) for RNA ligand recognition [ 7, 8]. However, only RIG-I and MDA5 possess N-terminal tandem caspase recruitment domains (CARDs) that directly interact with mitochondrial antiviral signaling (MAVS) to activate downstream signaling [2]. LGP2 lacks the CARD sequences and therefore cannot transduce RNA detection signaling but is thought to regulate RIG-I and MAD5 signaling pathways [4] .

RIG-I preferentially binds to blunt-end short (<300 bp) dsRNA that has a 5′-triphosphate (5′-PPP) moiety [ 9, 10]. MDA5 preferentially binds to longer dsRNA (>1000 bp) with no end specificity [4]. The tandem CARDs of RIG-I form contacts with the helicase domain and CTD when cells are free of viral infection [3]. Upon binding to viral dsRNA, RIG-I undergoes marked conformational rearrangements, oligomerization, and translocation to mitochondria, where it interacts with MAVS (also called VISA, IPS-1, or Cardif) via its CARDs [ 11‒ 14], resulting in the aggregation of MAVS to form a huge prion-like protein complex [15]. Next, several E3 ubiquitin ligases are recruited to the MAVS-associated complex, including TRAF2, 3, and 6, which also recruits TBK1 and the IKKα/β complex, leading to the activation of NF-κB and IRF3/7 [ 15, 16]. Activated NF-κB and IRF3/7 are translocated into the nucleus to induce transcription of downstream antiviral genes.

Posttranslational modifications (PTMs) are essential for the regulation of the RIG-I-mediated signaling pathway, particularly ubiquitin posttranslational modification [ 17 , 18]. There are seven different types of polyubiquitination linkages: K6, K11, K27, K29, K33, K48, and K63-linked ubiquitination. As mentioned above, K48- and K63-linked chain types have been well studied [19]. The K48-linked ubiquitin chains target protein substrates for proteasomal degradation, whereas the K63-linked modifications play nondegradative roles [18]. Several E3 ubiquitin ligases are critical for the regulation of RIG-I ubiquitination. TRIM25, a member of the tripartite motif (TRIM) protein family, is an E3 ligase that adds ubiquitin chains to RIG-I at lysine 172 within the second CARD [20]. TRIM4, another member of the TRIM family, also promotes K63-linked polyubiquitination of RIG-I at K154, K164, and K172 [21]. Another E3 ligase, MEX3C, also facilitates K63 polyubiquitination on K99 and K169 of the CARD [22]. K63-linked ubiquitination was confirmed for six RIG-I CARD lysine residues (K99, 169, 172, 181, 190, and 193) [20]. Other E3 ligases have been shown to regulate RIG ubiquitination, including Riplet which promotes K63-linked polyubiquitination of the CTD of RIG-I [23], and CBL, RNF122, and RNF125-mediated K48-polyubiquitination of RIG-I, leading to its proteasome-dependent degradation [ 24‒ 26] .

Zinc finger (ZNF) proteins are widespread and promote genome integrity. Zinc finger protein 205 (ZNF205) was first identified during the cloning of the gene for familial Mediterranean fever (FMF) [27]. Previous studies have reported that ZNF205 participates in the transcriptional regulation of human M-LP which is involved in the metabolism of reactive oxygen species [28]. Moreover, it has been reported that ZNF205-AS1 (ZNF205 antisense RNA 1) is associated with non-small cell lung cancer (NSCLC) [29]. However, little has been reported on the functions of ZNF205 in the regulation of innate immunity.

In the present study, we showed that ZNF205 is a positive regulator of the RIG-I-mediated antiviral signaling pathway and exerts functions by potentiating the K63-linked ubiquitination of RIG-I.

## Materials and Methods

### Antibodies and reagents

The antibodies used in this study is shown as follows: mouse anti-Flag (F3165; Sigma, St Louis, USA), anti-HA (H3663; Sigma), anti-Myc (sc-40; Santa Cruz Biotechnology, Dallas, USA), anti-IRF3 (sc-33641; Santa Cruz Biotechnology), anti-P65 and anti-phospho-P65 (#9936; Cell Signaling, Danvers, USA), anti-RIG-I (D14G6; Cell Signaling), rabbit anti-ZNF205 (CSB-PA026577XA01HU; Cusabio, Wuhan, China), HRP-conjugated anti-rabbit IgG Ab (H+L) (111-035-003; Jackson ImmunoResearch, West Grove, USA) and HRP-conjugated goat anti-mouse IgG Ab (172-1011; Bio-Rad, Hercules, USA). qPCR kit (A5001) was obtained from Promega (Madison, USA). SeV, VSV-GFP, and HSV-1 were kindly provided by Dr Hong-Bing Shu at Medical Research Institute of Wuhan University (Wuhan, China)

### Cell culture

293T, A549, MCF7, and Vero cells were kindly provided by Dr Hong-Bing Shu, and were grown in DMEM (Solarbio, Beijing, China) containing 10% fetal bovine serum (FBS; Cell Max, Beijing, China) with 1% penicillin and streptomycin (Solarbio) at 37°C in a 5% CO _2_ incubator.

### Plasmids

Mammalian expression plasmids for Flag- or HA-tagged human RIG-I, RIG-I-N (1‒284), ubiquitin and its mutants K48 or K63 ubiquitin, IFN-β promoter, and ISRE luciferase reporter plasmids were constructed as previously described [30]. Human Flag-, HA- or Myc-ZNF205 plasmids were constructed by standard molecular biology techniques, and the primers used were as follows: hZNF205-F- *Sal*I (forward): 5′-AAAGTCGACCATGTCTGCAGACGGCGGAGGC-3′; and hZNF205-R- *Not*I (reverse): 5′-AAAGCGGCCGCCTAGGTGGGAGCGGGTGGGGG-3′.

### Transfection and dual-luciferase reporter assay

293T cells were seeded in 24-well plates and transiently cotransfected with a firefly luciferase reporter gene (IFN-β-promoter or ISRE) and TK- *Renilla* luciferase reporter (internal control), together with the indicated plasmids or vector. Twelve hours later, cells were infected with Sendai viruses for the indicated time. After viral infection, luciferase activity was measured using a Dual-specific luciferase reporter kit (Promega). Luciferase assays were performed as previously described [31] .

### Quantitative RT-PCR

Total RNA from 293T cells transfected with the indicated plasmids was prepared using an RNA extraction kit (Promega) according to the manufacturer’s instructions. RNA (1 μg) was transcribed into cDNA using a reverse transcription kit (Promega). Real-time fluorescent quantitative PCR analyses were performed with a SYBR quantitative PCR kit (Promega). The relative mRNA levels of target genes in the samples were assessed by the comparative CT method and were normalized to *β-actin*. The primers of the target genes were as follows: *hβ-actin* forward: 5′-GTCGTCGACAACGGCTCCGGCATG-3′; *hβ-actin* reverse: 5′-ATTGTAGAAGGTGTGGTGCCAGAT-3′; *hIFNB1* forward: 5′-CTAACTGCAACCTTTCGAAGC-3′; *hIFNB1* reverse: 5′-GGAAAGAGCTGTAGTGGAGAAG-3′; and *hCXCL10* forward: 5′-GGTGAGAAGAGATGTCTGAATCC-3′; *hCXCL10* reverse: 5′-GTCCATCCTTGGAAGCACTGCA-3′.

### Plaque assay and viral infection

For RT-PCR or immunoblot analysis, cells were seeded into 24-well plates or 6-well plates and infected with Sendai viruses for the indicated time. For viral replication assays, cells were infected with VSV-GFP. After 1 h, the supernatants were cleared, and the cells were washed two times with prewarmed 1× PBS and then grown in complete medium. The replication of the virus was observed by fluorescence microscopy (Nikon, Tokyo, Japan). For the plaque assay, the supernatants were used to infect Vero cells. One hour later, the dilutions were removed, and the infected Vero cells were washed with prewarmed 1× PBS twice and then incubated with DMEM containing 2% methylcellulose for 48‒72 h. Cells were then fixed in 4% paraformaldehyde for 15 min and stained with 1% crystal violet for 20 min before plaque counting.

### Coimmunoprecipitation and immunoblotting and native PAGE

293T cells were seeded in 6-well plates and transfected with appropriate expression or blank control plasmids for 12 h, and then cells were infected with SeV for the indicated time. The infected cells were harvested and lysed using lysis buffer [1% Triton, 20 mM Tris-HCl pH 7.5, 150 mM NaCl, 1 mM EDTA, 1 mM phenylmethylsulfonyl fluoride (PMSF), 10 mg/mL aprotinin, and 10 mg/mL leupeptin]. The cellular lysates were prepared by centrifugation at 12,000 *g* for 8 min at 4°C. For each immunoprecipitation of protein, G/A-Sepharose beads (GE Healthcare, Piscataway, USA) were incubated with cellular lysates and appropriate amounts of the indicated antibodies overnight at 4°C. The coimmunoprecipitation assay and western blot analysis were performed as described previously [ 31, 32 ]. For the IRF3 dimerization assay, cells were treated with SeV or not for the indicated time and then collected and lysed in lysis buffer. Cellular extracts were dissolved in native PAGE sample buffer (62.5 mM Tris-Cl pH 6.8, 15% glycerol, and 1% deoxycholate) and analyzed by native PAGE as previously described [31].

### CRISPR-Cas9

To obtain the KO plasmid, the small guide RNAs (sgRNAs) of human ZNF205 were inserted into the lenti-CRISPR-V2 vector after annealing, which with psPAX2 and pMD2G was transfected into 293T cells. Supernatants were collected after 48 h and centrifuged at 800 *g* for 15 min. The lentivirus was used to infect HEK293 or A549 cells. The infected cells were screened with puromycin (1 μg/mL) for at least 5 days to obtain ZNF205-deficient cells.

### Fluorescence confocal microscopy

MCF7 cells were seeded on glass coverslips. After transfection, the cells were fixed with 4% paraformaldehyde for 15 min, permeabilized for 15 min with 0.2% Triton X-100 and blocked for 30 min with 1% bovine serum albumin (BSA) in PBS. Then, the cells were stained with the appropriate antibody. Nuclear staining was performed using DAPI (C0065; Solarbio). Fluorescence in cells was visualized with a Leica confocal microscope (Leica, Solms, Germany).

### Statistical analysis

All histogram data analysis was performed using GraphPad Prism (version 8.0; GraphPad Software, Inc., La Jolla, USA), and the values are the average and variance of three independent experiments. One-way ANOVA with Tukey’s post hoc analysis and two-way ANOVA statistical methods were used. *P* values less than 0.05 are considered statistically significant.

## Results

### ZNF205 interacts with and targets RIG-I

**Figure 1:**
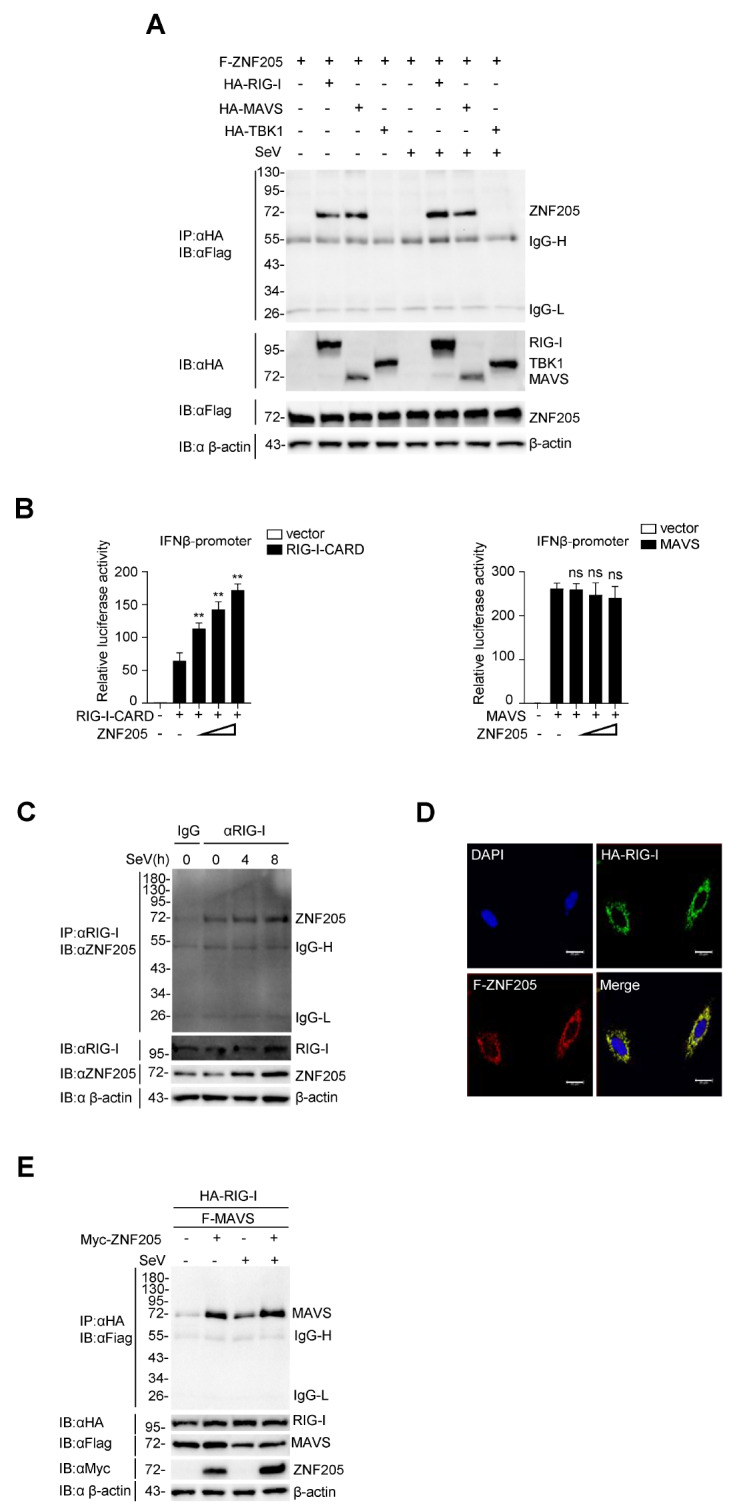
Figure 1 ZNF205 interacts with and targets RIG-I (A) ZNF205 interacts with RIG-I and MAVS. The indicated expression plasmids (2 μg each) were transfected into 293T cells (~2×106). Twelve hours after transfection, cells were uninfected or infected with SeV (MOI=1) for 12 h. Coimmunoprecipitation and immunoblot analyses were performed with the indicated antibodies. Western blot analysis was used to analyze the expression levels of the transfected proteins in the lysates. (B) ZNF205 inhibits SeV-induced innate immunity by targeting RIG-I. 293T cells (3×105) were transfected with the IFN-β promoter (50 ng), ZNF205 plasmids (0, 10, 100, or 500 ng), and RIG-I-CARD or MAVS for 22 h, and then subjected to luciferase analysis. (C) 293T cells (~2×107) were left uninfected or infected with SeV for 4 or 8 h. The lysates were immunoprecipitated with a control immunoglobulin G (IgG) or anti-RIG-I antibody, and immunoblot blotting was performed with the indicated antibodies. (D) MCF7 cells were cotransfected with Flag-ZNF205 and HA-RIG-I. Immunofluorescence microscopy was performed using anti-Flag (red) and anti-HA (green) antibodies. DAPI was used to stain the nuclei. Scale bar: 20 μm. (E) ZNF205 enhances the interaction between RIG-I and MAVS. ZNF205 or empty vector was cotransfected with RIG-I and MAVS expression plasmids (2 μg each) into 293T cells (~2×106). Twelve hours after transfection, cells were uninfected or infected with SeV (MOI=1) for 4 h. Coimmunoprecipitation and immunoblot analyses were performed with the indicated antibodies. Western blot analysis was used to analyze the expression levels of the transfected proteins in the lysates.

RIG-I plays a crucial role in the RLR antiviral signaling pathway. To identify additional regulators involved in RIG-I-mediated antiviral signaling, we performed a large-scale yeast two-hybrid screening assay using full-length RIG-I as a bait protein and found that ZNF205 is a candidate interactor of RIG-I [33]. We performed a coimmunoprecipitation assay in 293T cells to further confirm this result. ZNF205 interacted with RIG-I and MAVS ( Figure 1A). To further explore which protein ZNF205 targets, we overexpressed MAVS and RIG-I-CARD (aa1‒284) in 293T cells to test IFN-β production. The results showed that overexpression of ZNF205 increased RIG-I-CARD-mediated but not MAVS-mediated IFN-β promoter activation in a dose-dependent manner ( Figure 1B). Next, we performed endogenous coimmunoprecipitation experiments to further confirm the interaction between ZNF205 and RIG-1 protein, and the results showed that the interaction between ZNF205 and RIG-I was enhanced upon SeV infection ( Figure 1C). Meanwhile, immunofluorescence assays in MCF7 cells showed that ZNF205 was colocalized with RIG-I ( Figure 1D). In addition, we found that ZNF205 increased the interaction between RIG-I and MAVS ( Figure 1E). These results showed that ZNF205 interacts with and targets at RIG-I in the RLR antiviral signaling pathway.

### ZNF205 positively regulates RNA virus-induced IFN-β signaling

**Figure 2:**
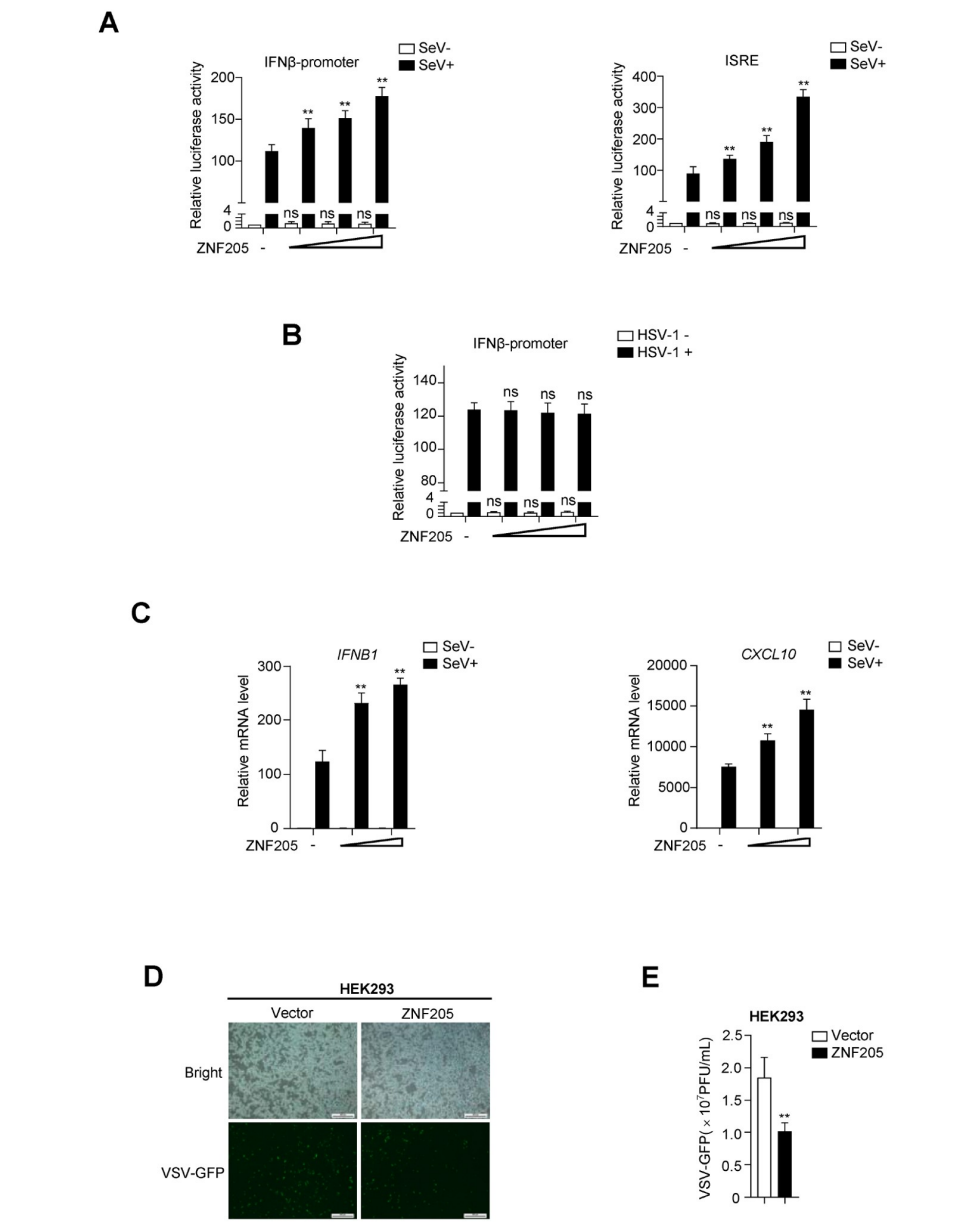
Figure 2 ZNF205 positively regulates the RIG-I-MAVS antiviral signaling pathway (A) ZNF205 enhances the SeV-mediated activation of the IFN-β promoter and ISRE. 293T cells were transfected with increasing concentrations of ZNF205 plasmids (0, 10, 100, or 500 ng) within IFN-β promoter (50 ng) or ISRE (50 ng) reporter plasmids. Twelve hours after transfection, cells were treated with SeV (MOI=1) for 12 h or left untreated, and reporter gene activity was then assayed using a luciferase kit. (B) ZNF205 had no significant effect on the HSV-1-induced IFN-β promoter. 293T cells were transfected with increasing concentrations of ZNF205 plasmids (0, 10, 100, or 500 ng) within IFN-β promoter (50 ng) reporter plasmids. Twelve hours after transfection, cells were treated with HSV-1 (MOI=1) for 10 h or left untreated, and reporter gene activity was then assayed using a luciferase kit. (C) Transcription of downstream antiviral genes is increased by overexpression of ZNF205. Control or ZNF205 plasmid was transfected into 293T cells. After 12 h of transfection, qPCR analysis was performed on cells left uninfected or infected with SeV for 12 h. Data are presented as the mean±SD, n=3. *P<0.05; **P<0.01; ns, no significant difference. (D) Effect of ZNF205 overexpression on VSV-GFP replication. HEK293 cells were transfected with control or ZNF205 (0.3 μg) plasmid. After 12 h of transfection, the cells were infected with VSV-GFP (MOI=0.1) for 11 h, and the cells were examined by fluorescence microscopy to detect VSV-GFP replication. (E) Overexpression of ZNF205 suppresses the replication of VSV-GFP. HEK293 cells were transfected with an empty vector or ZNF205 (0.3 μg) plasmid, and after 12 h of transfection, the cells were infected with VSV-GFP (MOI=0.1) for 12 h. Then, supernatants were collected and tested for viral titer.

To further investigate the role of ZNF205 in RNA virus-triggered innate immunity, we performed a dual-luciferase reporter assay, and the results showed that ZNF205 overexpression increased SeV-induced IFN-β promoter and ISRE luciferase reporter activation in a dose-dependent manner ( Figure 2A) but not HSV-1-induced IFN-β promoter activation ( Figure 2B). Consistently, real-time PCR also demonstrated that overexpression of *ZNF205* could potentiate SeV-induced transcription of downstream genes, including *IFNB1* and *CXCL10* ( Figure 2C). In addition, the replication of VSV-GFP was inhibited in HEK293 cells overexpressing ZNF205, as shown by GFP intensity and plaque assays ( Figure 2D,E). The above experiments showed that ZNF205 is a positive regulator of the RNA virus-induced antiviral signaling pathway.

### Knockdown of *ZNF205* impairs the RIG-I-MAVS antiviral signaling pathway

**Figure 3:**
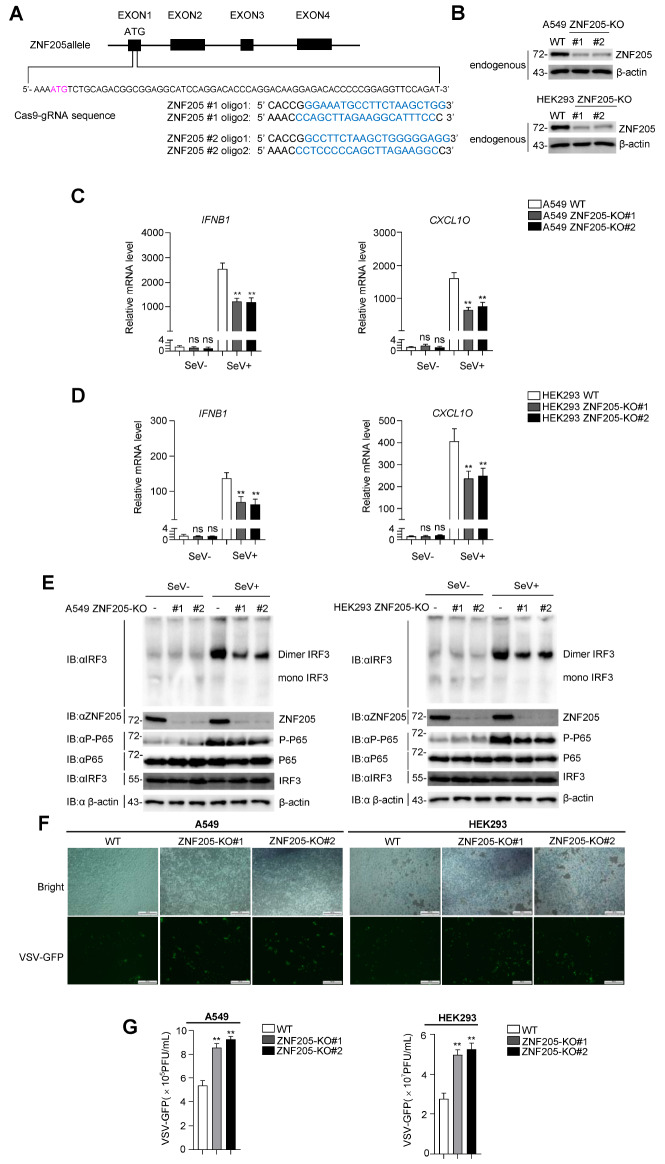
Figure 3 Knockdown of *ZNF205* impairs the RIG-I-MAVS antiviral signaling pathway (A) A schematic of CRISPR/Cas9-mediated genome editing of the ZNF205 gene locus. (B) ZNF205 knockout efficiencies. ZNF205 protein expression in ZNF205-deficient HEK293 and A549 cells compared to control cells determined by western blot analysis. (C,D) ZNF205 deficiency increases downstream antiviral gene transcription. qPCR analysis of ZNF205-deficient HEK293 and A549 cells left untreated or treated with SeV (MOI=1). (E) ZNF205 deficiency enhances SeV-induced dimerization of IRF3 and P65 phosphorylation. Immunoblot analysis of ZNF205-deficient A549 and HEK293 cells that were uninfected or infected with SeV (MOI=1). (F) Impact of ZNF205 deficiency on VSV-GFP replication. ZNF205-deficient A549 and HEK293 cells and control cells were treated with VSV-GFP at an MOI of 0.01, followed by phase contrast and fluorescence microscopy analyses. (G) ZNF205 deficiency in A549 and HEK293 cells potentiates the antiviral response. ZNF205-deficient A549 and 293T cells were treated with VSV-GFP (MOI=0.1). Supernatants were collected for plaque assays to determine virus titer.

Since IFN-β signaling is essential for host defense against viral infection, we further evaluated the influence of ZNF205 deficiency on viral infection and replication. We generated two different mixed ZNF205-deficient HEK293 and A549 cell lines (KO#1 and KO#2) using CRISPR-Cas9-mediated gene editing technology, and the effect of knockout was confirmed by western blot analysis ( Figure 3A,B). Real-time PCR assays revealed that the expression levels of *IFNB1* and *CXCL10* were inhibited by SeV. The result was consistent in ZNF205-deficient HEK293 and A549 cell lines ( Figure 3C,D). IRF3 dimerization and P65 phosphorylation after SeV infection were downregulated in ZNF205-deficient HEK293 and A549 cell lines ( Figure 3E). In addition, compared with its wild-type cells, we found that VSV-GFP replication was increased in ZNF205-deficient HEK293 and A549 cell lines by GFP intensities and plaque assay ( Figure 3F,G). These results suggested that ZNF205 deficiency inhibits the RLR-MAVS antiviral innate immune signal.

### ZNF205 functions through its zinc finger domain

**Figure 4:**
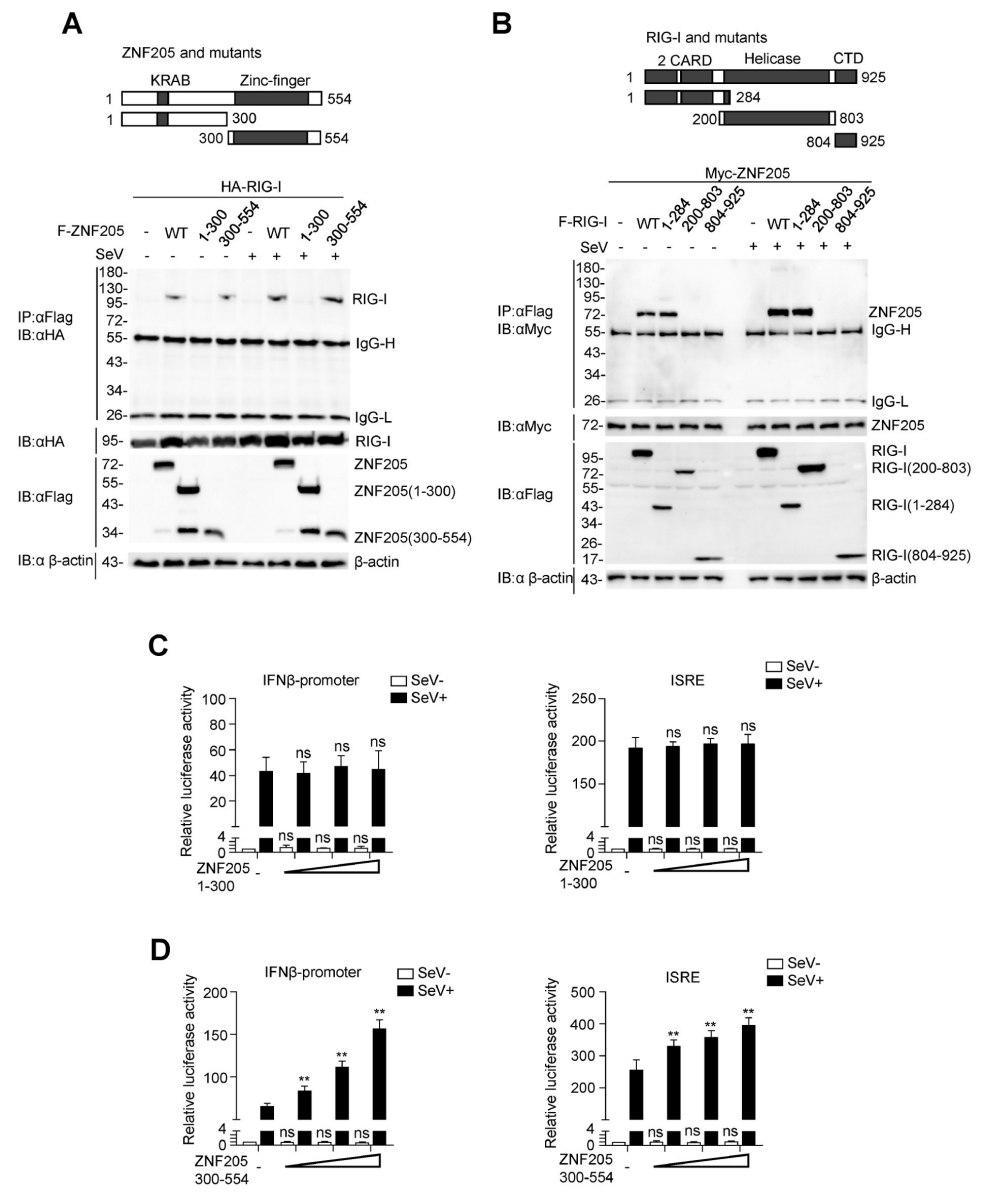
Figure 4 ZNF205 regulates RIG-I through the zinc finger domain (A,B) Immunoblot analysis of 293T cells transfected with plasmids encoding HA-RIG-I and FLAG-tagged ZNF205 or mutants (C) or plasmids encoding myc-ZNF205 and FLAG-tagged RIG-I or truncates (D), and coimmunoprecipitation and immunoblot analysis were performed with the indicated antibodies. (C) ZNF205 aa 1‒300 does not affect the SeV-mediated activation of the IFN-β promoter and ISRE. 293T cells were transfected with ZNF205 plasmids (0, 10, 100, or 500 ng) within IFN-β-promoter (50 ng) or ISRE (50 ng) reporter plasmids. Twelve hours after transfection, cells were treated with SeV (MOI=1) for 12 h or untreated, and reporter gene activity was then assayed using a luciferase kit. (D) ZNF205 aa 300‒554 increased the SeV-mediated activation of the IFN-β promoter and ISRE. 293T cells were treated similarly to Figure 4C.

The ZNF205 protein N-terminus contains a Krüppel-associated box (KRAB) domain, and the C-terminus contains a zinc finger domain. There are eight C2H2-type zinc finger motifs in the tandem array from residues 260 to 476 [27]. Domain mapping analyses suggested that the N-terminal CARD of RIG-I (aa1‒284) and C-terminal zinc finger domain of ZNF205 (aa300‒554) were responsible for their associations ( Figure 4A,B). Furthermore, we overexpressed ZNF205 (aa1‒300) and ZNF205 (aa300‒554) in 293T cells to perform a luciferase assay and found that ZNF205 (aa300‒554) increased the activation of the IFN-β promoter and ISRE in a dose-dependent manner. However, overexpression of ZNF205 (aa1‒300) had no effect ( Figure 4C,D). These results suggested that ZNF205 regulates RLR signaling by interacting with RIG-I through its zinc finger domain.

### ZNF205 promotes the K63-linked polyubiquitination of RIG-I

**Figure 5:**
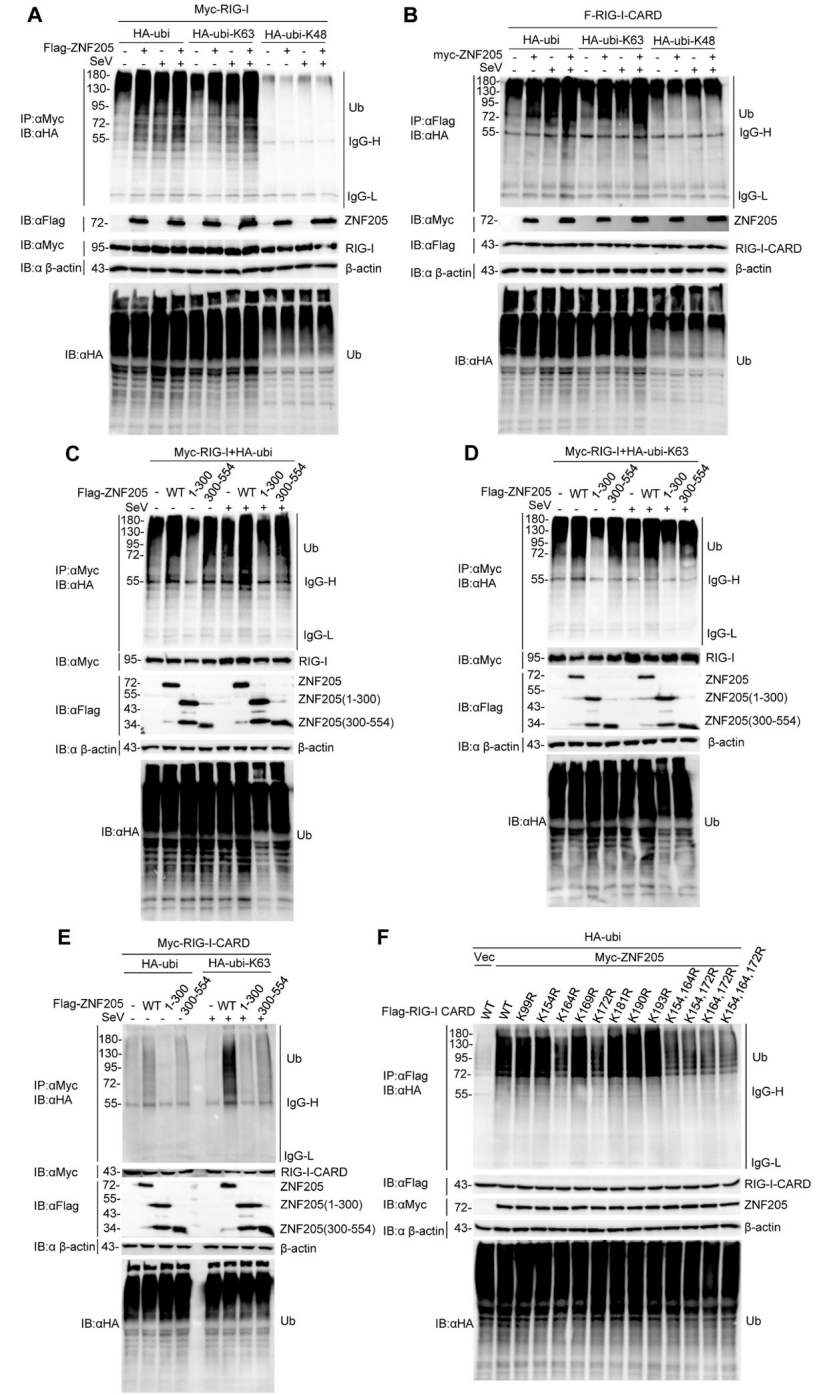
Figure 5 ZNF205 promotes the K63-linked polyubiquitination of MAVS (A) Overexpression of ZNF205 increases RIG-I polyubiquitination and K63-linked polyubiquitination. 293T cells (~2×106) were transfected with Myc-RIG-I, HA-tagged Ubi, Ubi-K48, Ubi-K63, and Flag-ZNF205 plasmids for 12 h, and then the cells were stimulated with or without SeV for 12 h, followed by coimmunoprecipitation and western blot analysis. (B) Polyubiquitination and K63-linked polyubiquitination of the CARD of RIG-I is increased by overexpression of ZNF205. 293T cells (~2×106) were transfected with Flag-RIG-I-CARD, HA-tagged Ubi, Ubi-K48, Ubi-K63, and Myc-ZNF205 plasmids for 12 h, and the cells were stimulated with or without SeV for 12 h, followed by coimmunoprecipitation and western blot analysis. (C) Overexpression of ZNF205 aa 300-554 increases the polyubiquitination of RIG-I. 293T cells (~2×106) were transfected with Myc-RIG-I, HA-ubiquitin (WT), and Flag-ZNF205 WT, aa1‒300 and aa 300‒554 plasmids for 12 h, and then cells were stimulated with or without SeV for 12 h, followed by coimmunoprecipitation and western blot analysis. (D) Overexpression of ZNF205 aa 300‒554 increases the K63-linked polyubiquitination of RIG-I. 293T cells (~2×106) were transfected with Myc-RIG-I, HA-ubiquitin (K63), and Flag-ZNF205 WT, aa1‒300 and aa 300‒554 plasmids for 12 h, and then cells were stimulated with or without SeV for 12 h, followed by coimmunoprecipitation and western blot analysis. (E) Overexpression of ZNF205 aa 300‒554 increases the K63-linked polyubiquitination of the CARD of RIG-I. 293T cells (~2×106) were transfected with Myc-RIG-I CARD, HA-ubiquitin (WT or K63), and Flag-ZNF205 WT, aa1‒300 and aa 300‒554 plasmids for 12 h, and cells were stimulated with or without SeV for 12 h and then subjected to coimmunoprecipitation and western blot analysis. (F) Overexpression of ZNF205 increases the polyubiquitination of the CARD of RIG-I at K164 and K172. 293T cells (~2×106) were transfected with F-RIG-I CARD (WT and mutants), HA-ubiquitin, and Myc-ZNF205 plasmids for 22 h, followed by coimmunoprecipitation and western blot analysis.

To further explore the molecular mechanism underlying the interaction of ZNF205 with RIG-I, we speculated that ZNF205 might participate in ubiquitin regulation of RIG-I, which is essential for its activation in antiviral responses [34]. To confirm this speculation, we performed a coimmunoprecipitation assay. The results showed that ZNF205 promoted ubiquitination and K63-linked ubiquitination of RIG-I but did not affect K48-linked ubiquitination of RIG-I ( Figure 5A). Since there are many locations of RIG-I ubiquitination on the CARD of RIG-I [ 20 ‒ 22], we performed coimmunoprecipitation again using RIG-I-CARD (aa1‒284), and the results demonstrated that ZNF205 promoted ubiquitination and K63-linked ubiquitination of RIG-I through the CARD of RIG-I ( Figure 5B). Next, we demonstrated that ZNF205 (aa300‒554), but not ZNF205 (aa1‒300), promoted ubiquitination and K63-linked ubiquitination of RIG-I ( Figure 5C,D). Then, we further demonstrated that ZNF205 regulates the ubiquitination and K63-linked ubiquitination of RIG-I via the interaction between ZNF205 (aa300‒554) and the CARD ( Figure 5E). These results suggested that ZNF205 targets the CARD domain of RIG-I to facilitate its ubiquitination. It has been demonstrated that some residues are associated with the ubiquitination of RIG-I, including K99, K154, K164, K169, K172, K181, K190, and K193 [ 20, 21]. We mutated the lysine residues mentioned above individually in the CARD domain of RIG-I to arginine. Moreover, we performed combined mutation of some residues, and we found that the ubiquitination of RIG-I was inhibited at K164 and K172, and the combined mutation contained K164 and K172 ( Figure 5F). These data showed the influence of ZNF205 on RIG-I K164 and K172 residues.

## Discussion

ZNF205 is a zinc finger protein. Zinc finger proteins are one of the most abundant groups of proteins in the human genome [35]. While ZNFs are best known as DNA-binding domains, it has become clear that ZNF domains can bind with RNA, lipids, and methylated DNA, as well as proteins and PTMs such as SUMO, ubiquitin, PAR, and methylation [36]. A few ZNF proteins have been reported to be associated with viral infection [ 37 , 38]. Among the reports already available about ZNF205, no report is relevant to viral infection.

In this study, we identified ZNF205 as a positive regulator of the modulation of IFN-β production by RIG-I. Overexpression of ZNF205 increased SeV-induced activation of the IFN-β promoter and the ISRE luciferase reporter and activation of transcription of downstream genes, such as *IFNB1* and *CXCL10*. The opposite result was obtained after the knockdown of *ZNF205*. These results showed that ZNF205 positively regulates the induction of IFN-β and the cellular antiviral response induced by SeV.

RIG-I is a critical cytoplasmic sensor for viral RNA, and its activation is strictly regulated by PTMs, especially ubiquitination. The binding of K63 polyubiquitin chains leads to the tetramerization of the RIG-I 2CARD, which is highly potent in activating MAVS and the downstream pathway [ 39, 40]. The RIG-I tetramer serves as a template to recruit individual CARDs of MAVS, leading to the assembly of MAVS into active, prion-like filaments [41]. In this study, we found that ZNF205 interacts with the CARD of RIG-I through its zinc-finger domain and enhances the interaction between RIG-I and MAVS.

Furthermore, we found that ZNF205 increases K63 ubiquitination of RIG-I CARD through its zinc-finger domain and that ZNF205 also targets RIG-I at K164 and K172 for K63-linked polyubiquitination. Previously, ZNF598 was reported to be an E3 ubiquitin ligase involved in the ribosome quality control pathway [42]. As a zinc finger protein, ZNF205 may also be an E3 ubiquitin ligase involved in the regulation of RIG-I. However, further experiments are needed to prove this speculation.

Several E3 ligases have been reported to regulate the K63 ubiquitination of RIG-I. TRIM25 is a ubiquitin ligase that interacts with the first CARD of RIG-I, and the K63-linked polyubiquitin moiety is then delivered to the K172 residue of the second CARD of RIG-I, resulting in efficient interaction with MAVS [ 20, 39]. The Riplet C-terminal region physically interacts with RIG-I CTD, and Riplet mediates K63-linked polyubiquitination of RIG-I CTD, leading to RIG-I activation [23]. The five lysine residues at 849, 851, 888, 907, and 909 on the CTD of RIG-I are essential for its polyubiquitination and activation [ 23, 40]. However, under some experimental conditions, Riplet binds to RIG-I CARDs [ 23, 41] to mediate K63-linked polyubiquitination of RIG-I CARDs at K154, K164, and K172 [42]. In addition to TRIM25 and Riplet, MEX3C and TRIM4 are also involved in the K63-associated polyubiquitination and activation of RIG-I [ 21, 22]. MEX3C mediates polyubiquitination at K99 and K169 [22], and TRIM4 targets K164 and K172 polyubiquitination of RIG-I [21]. ZNF205 also targets RIG-I at K164 and K172 for K63-linked polyubiquitination. It may also act as an E3 ligase-interacting protein, as described above, regulating its activity and thus indirectly regulating the K63 ubiquitination of RIG-I. However, how ZNF205 regulates the K63-linked polyubiquitination of RIG-I requires more profound research in the future.
